# Pyocyanin Facilitates Extracellular DNA Binding to *Pseudomonas aeruginosa* Influencing Cell Surface Properties and Aggregation

**DOI:** 10.1371/journal.pone.0058299

**Published:** 2013-03-11

**Authors:** Theerthankar Das, Samuel K. Kutty, Naresh Kumar, Mike Manefield

**Affiliations:** 1 Centre for Marine BioInnovation (CMB), School of Biotechnology and Biomolecular Sciences (BABS), University of New South Wales (UNSW), Sydney, Australia; 2 School of Chemistry, University of New South Wales (UNSW), Sydney, Australia; University of Technology Sydney, Australia

## Abstract

Pyocyanin is an electrochemically active metabolite produced by the human pathogen *Pseudomonas aeruginosa*. It is a recognized virulence factor and is involved in a variety of significant biological activities including gene expression, maintaining fitness of bacterial cells and biofilm formation. It is also recognized as an electron shuttle for bacterial respiration and as an antibacterial and antifungal agent. eDNA has also been demonstrated to be a major component in establishing *P. aeruginosa* biofilms. In this study we discovered that production of pyocyanin influences the binding of eDNA to *P. aeruginosa* PA14 cells, mediated through intercalation of pyocyanin with eDNA. *P. aeruginosa* cell surface properties including cell size (hydrodynamic diameter), hydrophobicity and attractive surface energies were influenced by eDNA in the presence of pyocyanin, affecting physico-chemical interactions and promoting aggregation. A Δ*phz*A-G PA14 mutant, deficient in pyocynain production, could not bind with eDNA resulting in a reduction in hydrodynamic diameter, a decrease in hydrophobicity, repulsive physico-chemical interactions and reduction in aggregation in comparison to the wildtype strain. Removal of eDNA by DNase I treatment on the PA14 wildtype strain resulted in significant reduction in aggregation, cell surface hydrophobicity and size and an increase in repulsive physico-chemical interactions, similar to the level of the Δ*phz*A-G mutant. The cell surface properties of the Δ*phz*A-G mutant were not affected by DNase I treatment. Based on these findings we propose that pyocyanin intercalation with eDNA promotes cell-to-cell interactions in *P. aeruginosa* cells by influencing their cell surface properties and physico-chemical interactions.

## Introduction

Planktonic bacterial cells tend to adhere, aggregate, colonize and grow into a mature biofilm on natural and synthetic surfaces. Biofilm formation is a serious ongoing problem ranging from lethal bacterial infections in humans, contamination of environmental resources such as water and corrosion of engineered systems [1]–[3]. Bacterial cells in biofilms are embedded in a matrix of extracellular polymeric substances (EPS) mainly composed of polysaccharides, proteins, lipids, metabolites and extracellular DNA (eDNA) [4], [5]. Extracellular DNA is recognised as a pivotal biofilm component in various Gram negative and Gram positive bacterial species. For instance in the opportunistic human pathogen *Pseudomonas aeruginosa* eDNA is essential for biofilm formation, stability and protection against antibiotics [6], [7]. In *Streptococcus mutans*, *Staphylococcus epidermidis* and *Bacillus cereus*, eDNA is essential for initial bacterial adhesion to surfaces. In *Staphylococcus aureus* eDNA enhances cellular aggregation and protects bacterial cells in biofilms against antibiotics and detergents [8]–[12].

In Gram positive bacteria quorum sensing (QS) dependent release of eDNA occurs through lysis of a small proportion of bacterial cells, mediated by proteins such as AtlE an autolysin produced by *S. epidermidis* [13], gelatinase (GelE) and serine protease (SprE) by *Enterococcus faecalis* [14]. In addition phage mediated and H_2_O_2_ mediated eDNA release as a consequence of cell lysis have also been reported in various *Streptococcus* species [15], [16]. However in Gram negative bacteria like *P. aeruginosa,* eDNA release is mediated through diverse mechanism. Quorum sensing involving *N*-acyl-L-homoserine lactones (AHL) and the *Pseudomonas* quinolone signal (PQS) activate eDNA release during late log phase in planktonic culture growth by inducing phage production [17]. QS independent mechanisms also cause cell lysis and subsequent eDNA release via flagella and type IV pili [17], [18]. Interestingly, it was recently discovered that eDNA release in *P. aeruginosa* also occurs through oxidative stress caused by hydrogen peroxide (H_2_O_2_) generation mediated by pyocyanin production [19].

Pyocyanin is a phenazine molecule produced abundantly by *P. aeruginosa* and is also controlled by QS [20]. The secondary QS molecule PQS controls the production of the *phzA-G* operons responsible for synthesis of the primary phenazine-1-carboxylic acid (PCA). PCA in *P. aeruginosa* PA14 strains is then modified to produce pyocyanin through action of the *phzM* gene product [21]. Pyocyanin controls gene expression and community behavior in divergent bacteria and colony size and biofilm thickness in *P. aeruginosa* PA14 [20], [22], [23]. Pyocyanin is electrochemically active and acts as an electron shuttle supporting *P. aeruginosa* cellular respiration and energy generation under oxygen deficient condition in biofilms, by accepting electrons for reoxidation of accumulating NADH [20]. Pyocyanin accepts electrons directly from NADH or NADPH and reduces molecular oxygen, forming reactive oxygen species like O_2_^−^ and H_2_O_2_. Reactive oxygen species change the redox balance of host cells causing cell injury and death [20] and hence pyocyanin is recognized as a virulence factor in chronic lung infections in cystic fibrosis patients [20].

Based on the facts that pyocyanin can act as an electron shuttle and eDNA promotes bacterial aggregation through acid-base interactions involving electron-donating and electron-accepting groups [9] we hypothesised that pyocyanin is involved in facilitating eDNA binding to *P. aeruginosa* cells. Further, we predicted that pyocyanin in association with eDNA could influence, *P. aeruginosa* cell surface properties such as size, hydrophobicity, surface energies and consequently influence cell-to-cell interactions (aggregation).

We tested our hypothesis in aggregation assays using *P. aeruginosa* PA14 wildtype, DKN370 (pyocyanin overproducing) and a Δ*phz*A-G (phenazine/pyocyanin deficient) mutants before and after removal of eDNA and in the presence of exogenous DNA (only for wildtype and Δ*phz*A-G strains). The binding/intercalation of pyocyanin with DNA was subsequently demonstrated directly. The surface properties of wildtype and Δ*phz*A-G strains before and after removal of eDNA were examined using techniques such as the Zetasizer to measure hydrodynamic diameter (size), goniometry for measuring hydrophobicity and thermodynamic analysis based on surface energies obtained from contact angles to reveal a possible mechanism of pyocyanin associated eDNA mediated aggregation in *P. aeruginosa* PA14.

## Materials and Methods

### *P. aeruginosa* Species and Culture Conditions

*P. aeruginosa* PA14 wildtype, DKN370 and Δ*phz*A-G (Note: The DKN370 and Δ*phz*A-G mutants was obtained from Dianne K. Newman) [22], [23] strains used in this study were plated onto LB agar plates and incubated overnight under aerobic conditions at 37°C. Single colonies from the agar plates were used to inoculate 20 ml cultures in LB medium in 100 ml conical flasks at ph 7 for 1, 2 or 3 days at 30°C and 150 rpm. After growth, the *P. aeruginosa* PA14 strains were harvested and pelleted out by centrifugation at 5000×g for 5 min at 10°C. Where indicated the pyocyanin deficient strain Δ*phz*A-G *was* also grown in the presence of 100 µm pyocyanin (Sigma-Aldrich) for 1, 2 and 3 days at 30°C, 150 rpm. To remove naturally present eDNA from the *P. aeruginosa* cell surfaces, bacterial suspensions were pre-treated with 40 units (2 units/µl) of DNase I (Life technologies) in the presence of 5 mM MgCl_2_ for 90 min at room temperature under static conditions and subsequently washed twice with phosphate buffered saline (PBS) and resuspended in PBS.

### Aggregation and Settling of *P. aeruginosa* PA14 before and after DNase I Treatment

To study *P. aeruginosa* PA14 aggregation before and after DNase I treatment, bacterial cells were diluted to 2.5×10^8^ cells/ml in PBS. Optical density was determined in 1 ml plastic cuvettes (SARSTEDT) using a Bio-Rad Smartspec 3000 (Bio-Rad Laboratories, USA) at 600 nm. The bacteria were allowed to aggregate and settle for 60 min at room temperature in plastic cuvettes under static conditions while monitoring the decrease in optical density (OD) every 15 min, using a Bio-Rad Smartspec 3000. Settling as a consequence of aggregation was quantified as a percentage reduction in OD after 60 min [24]

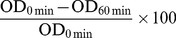
(1)where OD_0min_ is the initial OD at the start of an experiment and OD_60min_ is the OD after 60 min. All measurements were done at 600 nm using PBS without bacteria as blank.

### Chromosomal DNA Isolation from *P. aeruginosa* PA14

Chromosomal DNA was extracted from a one day old culture of *P. aeruginosa* PA14 using standard protocols [9]. The extracted DNA was then dissolved in MilliQ water and the DNA concentration was determined using a Nanodrop UV/Vis spectrophotometer. Purity of the DNA was determined by the absorbance ratio A_260_/A_280_ (DNA/protein)_._ All DNA samples used in this study have A_260_/A_280_ equal to 1.8, indicating a high purity.

### Aggregation and Settling of *P. aeruginosa* PA14 in the Presence of Exogenous DNA

A total of 2.25×10^8^ cells in 900 µl PBS were mixed with 100 µl of a DNA solution or 100 µl MilliQ water as a negative control in 1 ml plastic cuvettes by pipetting. The final concentration of DNA present was 1 µg/ml. The settling of bacterial cells due to aggregation mediated by the presence or absence of exogenous DNA was monitored every 15 min.

### Hydrodynamic Diameter of *P. aeruginosa* PA14

The hydrodynamic diameter of *P. aeruginosa* PA14 wildtype and Δ*phz*A-G strains of cell density 2.5×10^8^ cells/ml before and after DNase I treatment was measured in PBS using a Zetasizer (nano series Model Number ZEN3600) (Malvern Instruments Ltd) at 25°C. Backscattered light from a monochromatic laser (633 nm wavelength) after passing through the bacterial cell suspension was detected at an angle of 173°. Subsequently, the autocorrelation of the time-dependent intensity fluctuations of the scattered light were used to derive the Brownian motion velocity of the bacteria, from which the hydrodynamic diameter was then calculated using the Stokes-Einstein equation [25].

### Pyocyanin-DNA Binding

Binding of pyocyanin to DNA was demonstrated using a standard ethidium bromide-DNA binding technique [26] using a fluorometer (Varian Cary Eclipse Fluorescence Spectrophotometer by Varian Australia Pvt. Ltd). DNA (4 ng/µl) was mixed with ethidium bromide (4 µM) and various concentrations of pyocyanin in SHE buffer (2 mM Hepes, 10 µM EDTA and 9.4 mM NaCl in MilliQ water adjusted to pH 7 with NaOH). Light emission at 615 nm (λ_ex_ = 480 nm) was quantified at room temperature.

To further visualize pyocyanin-DNA binding, DNA (2 mg) isolated from *P. aeruginosa* PA14 wildtype was mixed with pyocyanin at 500 µM (Sigma-Aldrich) at both acidic (4.5) and neutral pH by pipetting. At acidic pH pyocyanin appears pink, while at neutral or alkaline pH it appears blue. The DNA-pyocyanin solution was incubated static overnight at room temperature followed by centrifugation at 5000×*g* for 5 min at 10°C in order to pellet out the DNA from the pyocyanin solution. The supernatant was removed by pipetting and the DNA pellet was air-dried. The colour of DNA pellets was recorded using using a digital camera.

### Contact Angles Measurements and Surface Thermodynamical Analysis of *P. aeruginosa* PA14

For contact angle measurements, bacterial lawns were prepared by depositing bacteria from suspension on a 0.2 µm-pore-diameter filter (Nitrocellulose membrane filter, Millipore) using negative pressure [8]. The filters were air dried at room temperature until contact angles of sessile water droplets were stable (30–45 min). Contact angles were measured with standard polar and non-polar liquids i.e. water, formamide and diiodomethane respectively. Where indicated a bacterial lawn of DNase I treated *P. aeruginosa* was prepared and its contact angle was measured as above.

To conduct surface thermodynamical analysis, the measured contact angles of *P. aeruginosa* cell surface before and after DNase I treatment were converted into its Lifshitz-Van der Waals (γ^LW^) and acid-base (γ^AB^) surface free energy components using the LW-AB approach [27], [28]. Subsequently, the acid-base component was separated in an electron-donating (γ^−^) and electron–accepting (γ^+^) parameter [27]. Different components of the surface energies were then further used to calculate the total interfacial free energy of aggregation (Total ΔG) at close contact as separated in a Lifshitz-Van der Waals (LW ΔG) and an acid-base (LW ΔG) part [28].

### Statistical Analysis using Student t-test

The effects of naturally produced pyocyanin and DNase I treatment on *P. aeruginosa* PA14 aggregation, contact angle and surface thermodynamics were analyzed using a two-tailed Student’s t-test. A similar analysis was carried out on the effect of exogenous DNA addition on *P. aeruginosa* aggregation. Differences were considered significant if p<0.05.

## Results

### Pyocyanin Production and Effect of DNase I Treatment in Settling of *P. aeruginosa* Strains

**Figure 1:**
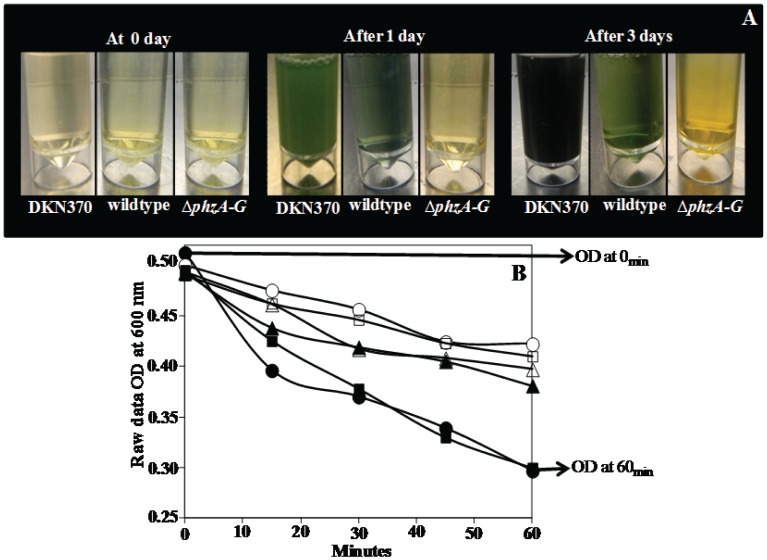
Pyocyanin production in *P. aeruginosa* PA14 strains and effect of DNase I treatment in PA14 aggregation. (A) Shows production of pyocyanin in planktonic culture of PA14 DKN370 and wildtype (indicated by green colour) and lack of pyocyanin production in Δ*phzA-G* strain even over 3 days growth period. (B) Represents an example of raw data showing decrease in absorbance at 600 nm OD due to aggregation of PA14 DKN370, wildtype and Δ*phzA-G* over 60 minutes. DKN370: before DNase I treatment (closed square), after DNase I treatment (open square), wildtype: before DNase I treatment (closed circle), after DNase I treatment (open circle), Δ*phzA-G*: before DNase I treatment (closed triangle), after DNase I treatment (open triangle).

In this study, *P. aeruginosa* PA14 cell surface properties and aggregation were investigated in the presence and absence of eDNA. The DKN370 and wildtype strain producing pyocyanin was compared with the pyocyanin deficient mutant strain Δ*phzA-G*. Figure 1A illustrates the visible difference between the three strains with respect to pyocyanin production. Figure 1B demonstrates the settling behaviour of the DKN370, wildtype and Δ*phzA-G* culture before and after DNase I treatment, due to aggregation, indicated by decreases in optical density at 600 nm over time.

### Quantification of Settling of DKN370, Wildtype and Δ*phzA-G* mutant of *P. aeruginosa* before and after DNase I Treatment

**Figure 2:**
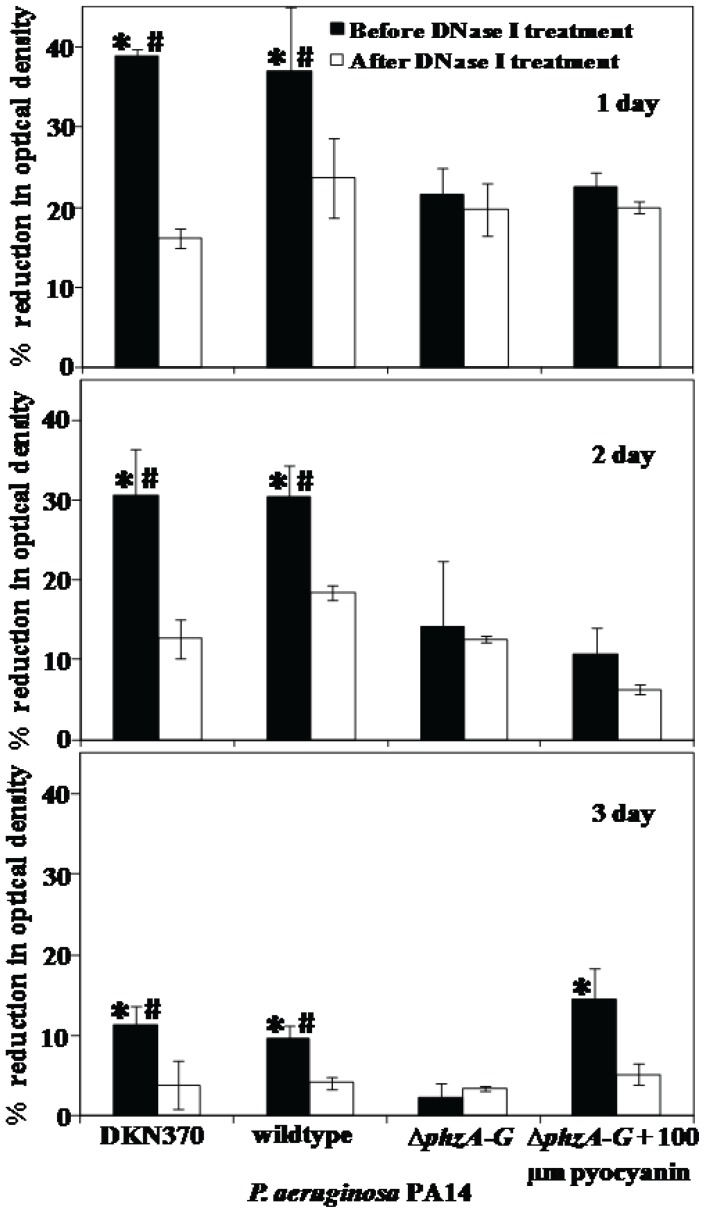
Influence of pyocyanin and DNase I treatment in aggregation of *P. aeruginosa* PA14. The % reduction in optical density after 60 min due to aggregation of PA14 strains before and after DNase I treatment. Error bars represents standard deviations from the mean (n = 3). Asterisks and hash indicate statistically significant (p<0.05) differences in % of aggregation in comparison to DNase I treated (DKN370 and wildtype) and Δ*phzA-G* strain (regardless of DNase I treatment) respectively.

Figure 2 shows that *P. aeruginosa* PA14 strains showed maximum settling up to 39%, 37% and 22% for the DKN370, wildtype and Δ*phzA-G* mutant respectively when grown over one day and before DNase I treatment. As the growth period increased the percentage of settled cells decreased drastically down to 12, 10 and 3% for DKN370, wildtype and Δ*phzA-G* respectively over 3 days of growth. Before DNase I treatment, settling of *P. aeruginosa* PA14 DKN370 and wildtype was always significantly higher in comparison to its pyocyanin deficient counterpart regardless of growth days. After DNase I treatment the settling percentage of the DKN370 and wildtype decreased significantly to a level equivalent to the Δ*phzA-G* mutant percentage. However, no difference in the Δ*phzA-G* settling percentage was observed when treated with DNase I. Interestingly when Δ*phzA-G* was grown with 100 µm pyocyanin a reduction in the settling percentage was observed after DNase I treatment although the difference was significant only after 3 days.

### Effect of DNA Addition in Settling of *P. aeruginosa*

**Figure 3:**
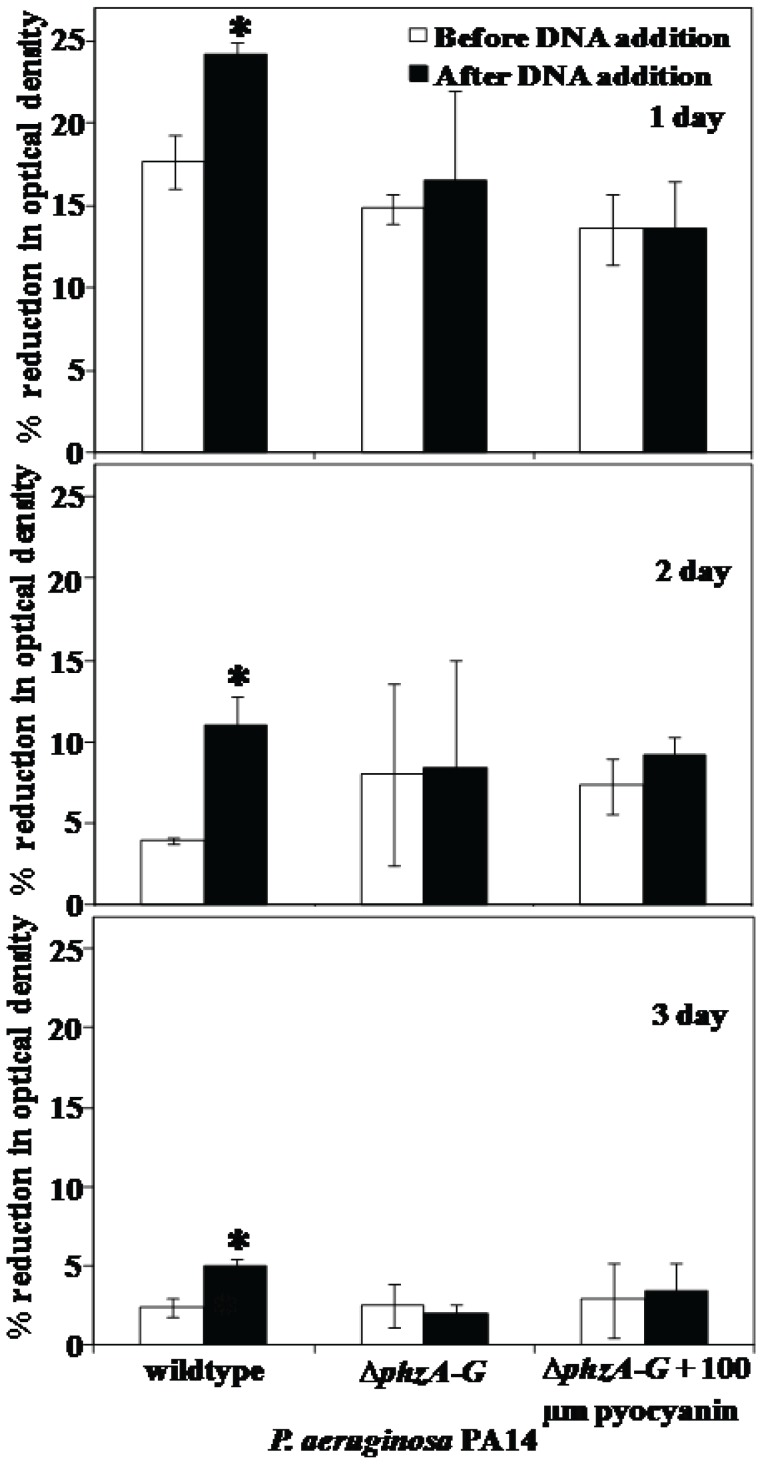
Effect of DNA addition in aggregation of *P. aeruginosa* PA14. The % reduction in optical density after 60 min due to aggregation of PA14 wildtype and Δ*phzA-G* strains before and after addition of 1 µg/ml of final concentration of exogenous DNA isolated from *P. aeruginosa* cultures. Error bars represents standard deviations from the mean (n = 3). Asterisks indicate statistically significant (p<0.05) differences in % of aggregation in comparison to DNase I treated wildtype strain.

Figure 3 shows that exogenous addition of 1 µg/ml DNA (isolated from *P. aeruginosa* cultures) to 2.25×10^8^ DNase I treated *P. aeruginosa* PA14 cells resulted in significant increases in the settling percentage of the wildtype strain only. The phenazine deficient mutant Δ*phzA-G* did not show any increase in percentage settling when treated with exogenous DNA. When the Δ*phzA-G* mutant was grown in the presence of exogenously added pyocyanin only a slight variation in settling percentage was observed, indicating endogenous production of eDNA, pyocyanin or both was required.

### Hydrodynamic Diameter (size) Analysis of *P. aeruginosa* Wildtype and Δ*phzA-G* Strains

**Figure 4:**
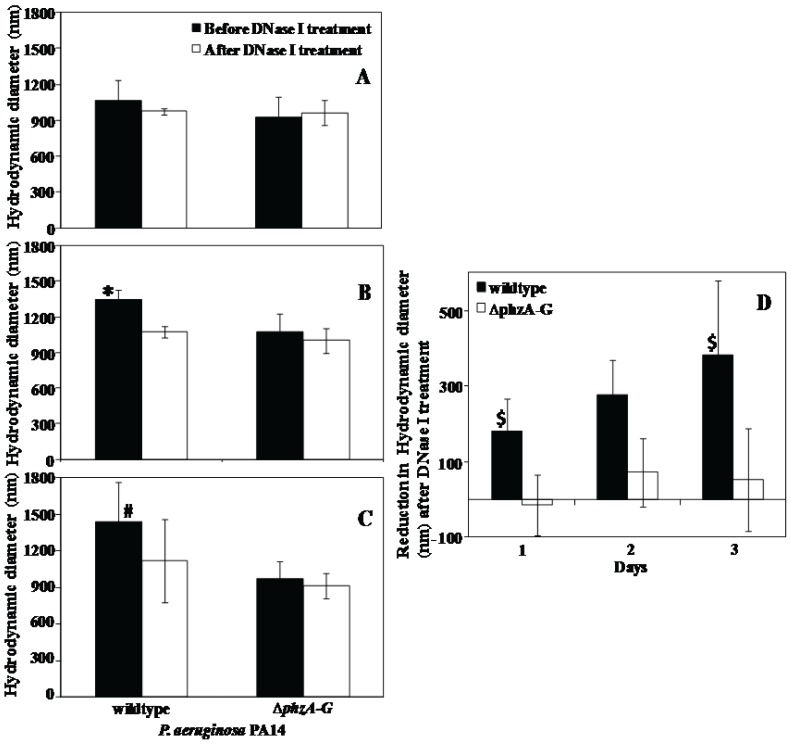
Size analysis of *P. aeruginosa* PA14 strains. (A to C) Hydrodynamic diameter of PA14 wildtype and Δ*phzA-G* strains, grown over 1 to 3 days respectively and effect of DNase I treatment on it. (D) Represents the change in reduction in hydrodynamic diameter of wildtype and Δ*phzA-G* after DNase I treatment. Error bars represents standard deviations from the mean (n = 5). Asterisks and hash indicate statistically significant (p<0.05) differences in hydrodynamic diameter in comparison to DNase I treated wildtype and Δ*phzA-G* strain (regardless of DNase I treatment) respectively. Dollar indicates statistically significant (p<0.05) differences in reduction in hydrodynamic diameter between wildtype (before and after DNase I treatment) and Δ*phzA-G* (before and after DNase I treatment) respectively.

The hydrodynamic diameter of *P. aeruginosa* PA14 wildtype before DNase I treatment showed a gradual increase in size from 1065±170 to 1440±300 nm with the increase in growth period from 1 to 3 days respectively. Whereas, the Δ*phzA-G* mutant showed a smaller variation in size (920±175 to 1050±160 nm) and remained in the same size range after DNase I treatment. In contrast, the wildtype strain showed a steady decrease in its size after DNase I treatment, ultimately close to the size of the Δ*phzA-G* mutant strain (Figure 4A–C). Figure 4D demonstrates clearly that after DNase I treatment the hydrodynamic diameter of the wildtype reduced to about 180±88 nm after 1 day growth to a maximum of 382±197 nm after 3 days of growth. In parallel, the mutant Δ*phzA-G* showed negligible reduction in its hydrodynamic diameter after DNase I treatment ranging from −15 to 70 nm only.

### Influence of Pyocyanin Production in Formation of EPS in *P. aeruginosa* Wildtype

**Figure 5:**
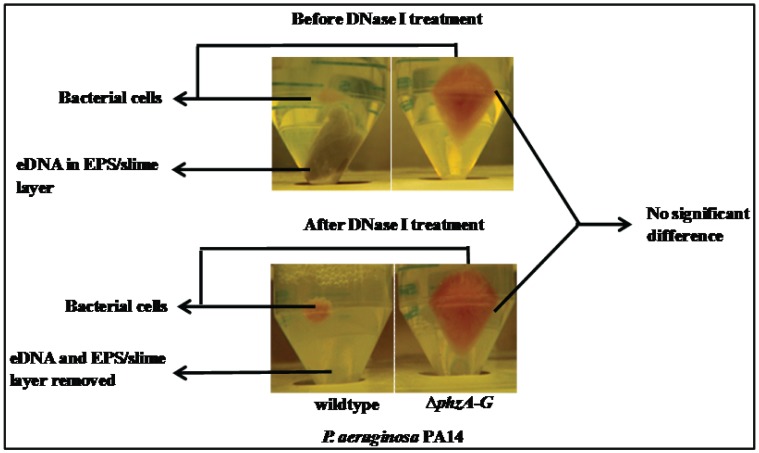
Pyocyanin binding with DNA responsible for thick slimy EPS formation. Photographic image showing formation of bacterial pellet and thick slimy eDNA constituted EPS (only on wildtype strain) formation after harvesting and centrifugation of 3 day old planktonic culture of *P. aeruginosa* PA14 strains (above panel). The slimy eDNA constituted EPS degraded completely after DNase I treatment in wildtype strain where as the pyocyanin deficient mutant Δ*phzA-G* does not show any effect on DNase I treatment (below panel).

Figure 5A shows the cell pellet formed after centrifugation of a 3 day old *P. aeruginosa* PA14 culture. The wildtype forms a very small pellet encased in EPS. In contrast the Δ*phzA-G* forms a larger pellet lacking EPS. The EPS in the wildtype pellet was removed when subjected to DNase I treatment whilst no difference was observed in the case of Δ*phzA-G* mutant after DNase I treatment (Fig. 5).

### Binding of Pyocyanin with DNA

**Figure 6:**
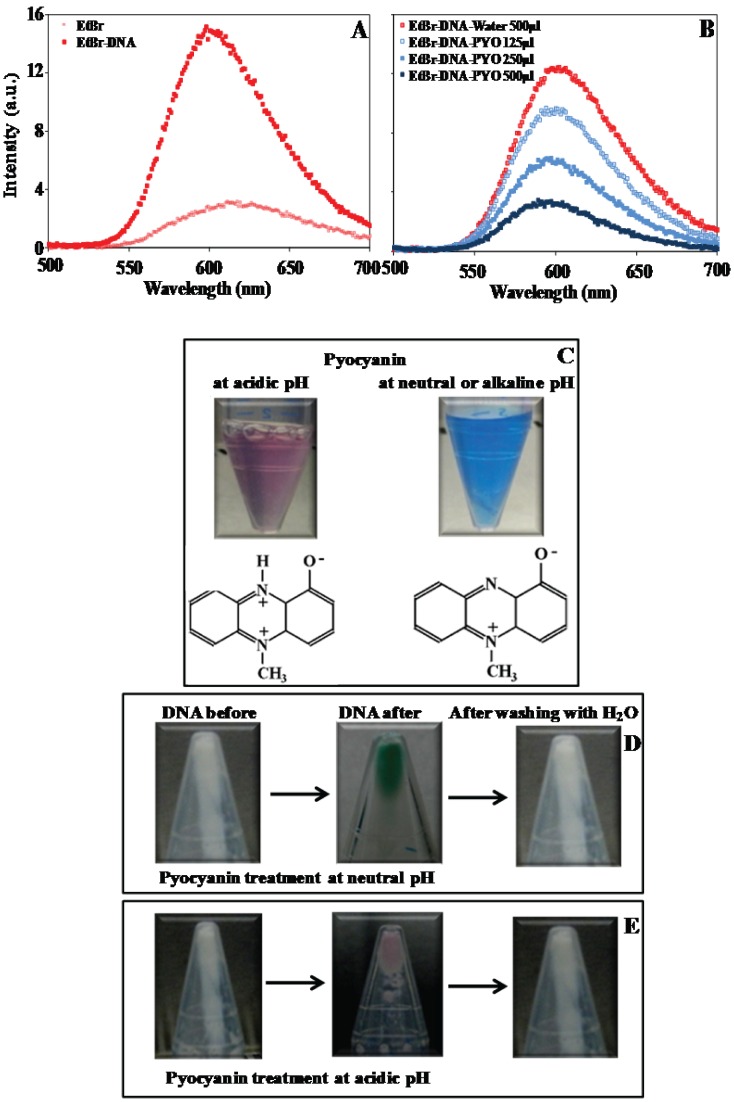
Pyocyanin intercalation with DNA. (A) Fluorescence emission spectra of ethidium bromide before and after DNA addition. (B) Decrease in intensity of emission spectra of ethidium bromide-DNA in presence and absence of pyocyanin. (C) Showing structure and colour of pyocyanin at acidic and neutral pH. (D and E) Showing colour change in DNA pellet from white to greenish blue in neutral pH and pink under acidic pH, after treated with pyocyanin and incubated at room temperature for overnight, indicates pyocyanin binded with DNA and the reappearance of white colour of DNA by simple washing of coloured DNA pellet with water resulted in removal of pyocyanin stain (Fig. 6D and E).

Figure 6A shows the relative fluorescence emission of ethidium bromide before and after addition of DNA. The initial intensity of the ethidium bromide emission peak which was only 3.2 rose gradually after addition of DNA as a function of concentration (data not shown) and reached 15.2 with 4 ng/µl DNA. The addition of 40–140 µM pyocyanin reduced the intensity of the fluorescence output indicative of displacement of ethidium bromide from DNA.

Pyocyanin binding with DNA was further confirmed by mixing DNA with synthetic pyocyanin and incubating overnight at room temperature. A change in the colour of DNA pellets from white to greenish blue in neutral pH and pink under acidic pH was observed. Washing the coloured DNA pellets with water removed the colour indicating that the binding of pyocyanin with DNA is reversible (Fig. 6D and E).

### Contact Angle and Subsequent Surface Energy Analysis of *P. aeruginosa* Strains before and after DNase I Treatment

Table 1 illustrates the changes in surface hydrophobicity and surface energy of one day old cultures of *P. aeruginosa* PA14 wildtype and mutant before and after DNase I treatment. Untreated PA14 wildtype showed a higher degree of water contact angle in comparison to untreated Δ*phzA-G*. After DNase I treatment only the wildtype strain showed a significant decrease in water contact angle and ultimately equivalent to the water contact angle of the Δ*phzA-G* mutant. The surface energy components: Lifshitz-Van der Waals remained unaffected between wildtype and Δ*phzA-G* regardless of DNase I treatment. However, the electron donating and electron accepting parameters of the acid-base component was significantly different between the wildtype and mutant strains in the absence of DNase I treatment.

**Table 1:** Contact angles of *P. aeruginosa* PA14 strains before and after DNase I treatment using water, formamide and diiodomethane and their surface free energy components: Lifshitz-Van der Waals (γ^LW^), acid-base (γ^AB^), electron-donating (γ^−^) and electron-accepting (γ^+^) based on their contact angle values.

Bacterial species	DNase Itreatment	Contact angle (θ) in ° (degrees)			Surface energy (mJ/m^2^)			
*P. aeruginosa* PA14		Water	Formamide	Diiodomethane	γLW (Lifshitz-Vander Waals)	AB (acid-base)		
						γ- (electrondonating)	γ+ (electronaccepting)	γAB
Wildtype	No	49.9±5.9*	39.4±0.8	49.1±1.2	34.7±0.6	27.9±6.1*	1.2±0.07*	11.3±0.9
wildtype	Yes	32.3±1.8	38.1±3.0	47±2.1	35.1±2.2	51.9±0.4	0.4±0.07	8.5**±**0.8
Δ*phzA-G*	No	36±1.7	43.3±2.4	44.1±6.9	37.4±3.6	52.2±0.4	0.1±0.14	3.2±4.5
Δ*phzA-G*	Yes	34.2±1.4	43.7±1.7	44.6±6.1	34±1.1	55.2±4.2	0.1±0.1	3.2±4.5

Error bars represents standard deviations from the mean (n = 3). Asterisks indicate statistically significant (p<0.05) differences in contact angle and surface energy in comparison to DNase I treated wildtype and Δ*phzA-G* strain regardless of DNase I treatment.

### Effect of Pyocyanin Production and DNase I Treatment on Interfacial Free Energy of Aggregation of *P. aeruginosa* Strains

**Figure 7:**
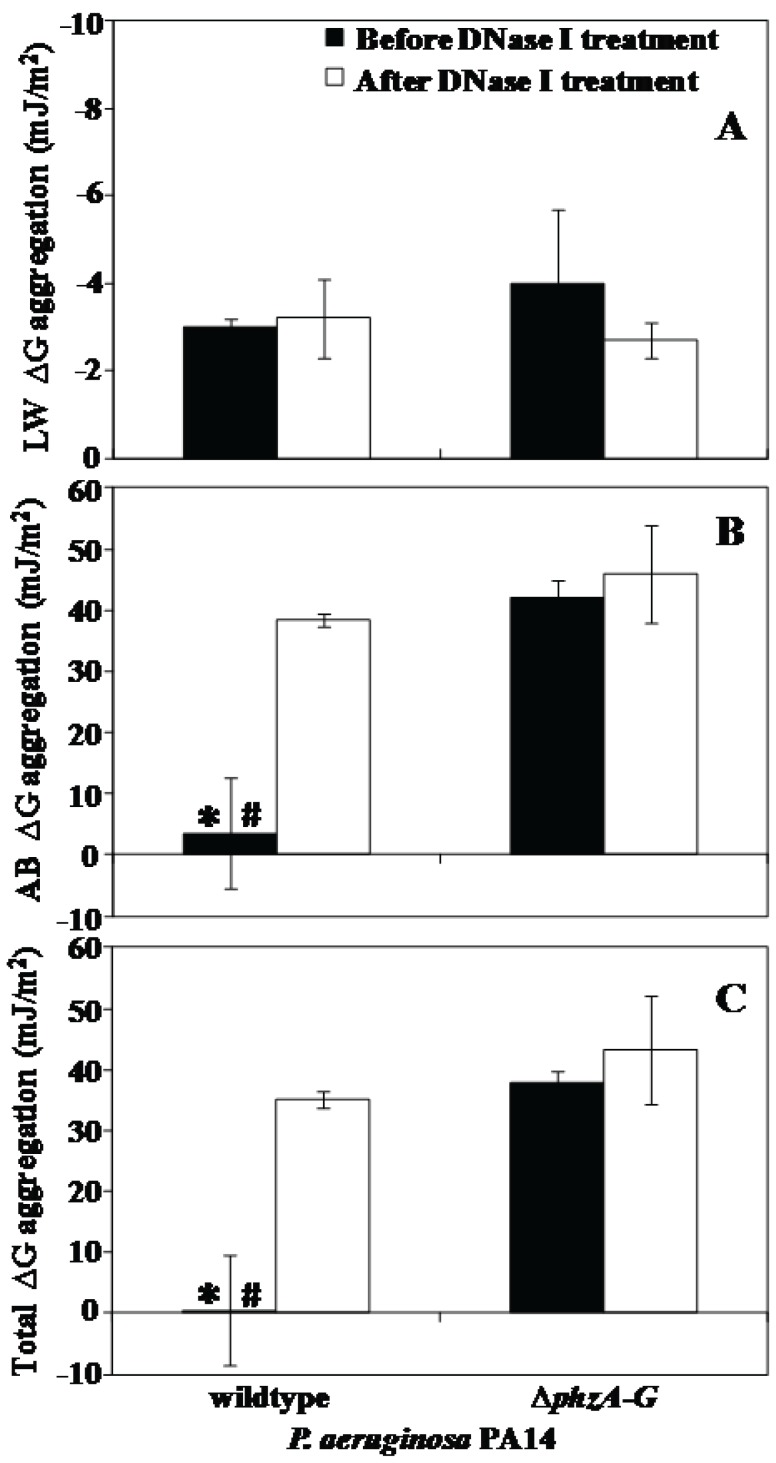
Interfacial free energy of aggregation of *P. aeruginosa* PA14 strains. (A to C) Components: Lifshitz-Van der Waals (LW ΔG) and acid-base (AB ΔG) and total interfacial free energy (Total ΔG) of aggregation of PA14 strains before and after DNase I treatment. Error bars represents standard deviations from the mean (n = 3). Asterisks and hash indicate statistically significant (p<0.05) differences in the free energy of aggregation in comparison to DNase I treated wildtype and Δ*phzA-G* strain regardless of DNase I treatment respectively.

Interfacial aggregation energies at close approach are presented in Figure 7. Lifshitz-Van der Waals aggregation energies were attractive for both strains, regardless of DNase I treatment (Figure 6A). The values for acid-base aggregation energies were very low 3 mJ/m^2^ for the wildtype strain before DNase I treatment and drastically high (40 mJ/m^2^) after DNase I treatment. In contrast, the acid-base energies of Δ*phzA-G* strain remained unchanged (45 mJ/m^2^) even after DNase I treatment (Fig. 7B). Similarly, the value for total aggregation energies is almost zero for the wildtype strain before DNase I treatment. After DNase I treatment the total aggregation energies for wildtype increased drastically, whereas the Δ*phzA-G* remained highly positive between 40 to 45 mJ/m^2^ regardless of DNase I treatment (Fig. 7C).

## Discussion

Pyocyanin and eDNA are both currently recognized as pivotal components in the development of *P. aeruginosa* biofilm [6], [20], [22]. Whilst the aggregation of bacteria is dependent of a variety of factors there is clear evidence that attachment of eDNA to cell surfaces influences physico-chemical interactions and facilitates aggregation [9]. There is also clear evidence that pyocyanin plays a role in eDNA release [19] and respiration under electron acceptor limited conditions [20]. Here we show for the first time that naturally produced pyocyanin promotes eDNA binding to *P. aeruginosa* cells and consequently influences cell surface properties, physico-chemical interaction between cells and aggregation.

In the presence of eDNA acid-base and total interaction energies represent an extremely low energy barrier that microbial cells can overcome through hydrogen bonding due to the presence of biopolymers and cell appendages that extend hundreds of nanometers from the cell surface [29]. In support of this, the removal of eDNA by DNase I treatment in this study, resulted in a drastic increase in the acid-base and total energy barrier to aggregation up to 14 and 70 fold respectively. Consequently the acid-base and total interaction energies become highly repulsive and bacterial aggregation decreased significantly (Fig. 2 and 7).

Interestingly, DNase I treatment did not have any effect on aggregation of a pyocyanin deficient mutant, which has a reduced ability to aggregate compared with the pyocyanin overproducing DKN370 and normal amount of pyocyanin producing wildtype (Fig. 2). It should be noted that DKN370 also showed a similar level of aggregation to its wildtype strain despite over production of pyocyanin (Fig. 2). It has been recently discovered that pyocyanin over production can enhance the release of eDNA [19] however binding of eDNA to bacterial cells is a limiting factor, once the bacterial cells surfaces are saturated with eDNA, further addition of DNA does not encourage further increase in adhesion or aggregation [9], [30]. Further, the acid-base and total interactions energies of pyocyanin deficient mutant remained unchanged and highly repulsive regardless of DNase I treatment (Fig. 7). However, DNase I treatment did affect aggregation of a mutant strain grown in the presence of exogenously added pyocyanin although the results are significant only on day 3 of culturing (Fig. 2). It could be due to the higher swarming motility of phenazine deficient mutants that makes them not responsive to exogenous pyocyanin at early stages of biofilm development [22]. However during later stages of biofilm growth they become responsive to pyocyanin and promote biofilm development [22]. In connection with the current study phenazine deficient mutants may possibly become responsive to exogenous pyocyanin only at 3 day culture and thus binds with eDNA and enhanced aggregation (Fig. 2). Additionally, when DNase I treated wildtype and Δ*phzA-G* mutant were exposed to exogenously added DNA the wildtype strain showed a significant increase in aggregation whilst the pyocyanin deficient mutant did not (Fig. 3). Taken together, these results are consistent with the hypothesis that pyocyanin facilitates cell aggregation through an interaction with eDNA.

A fluorescence assay, involving displacement of the DNA intercalating agent ethidium bromide from DNA, was used to confirm that pyocyanin binds directly to DNA. DNA intercalation occurs via noncovalent interactions and is reversible. In this study pyocyanin binding was reversible as shown by the decolourization of pyocyanin-DNA pellets after washing with water. DNA intercalating agents cause substantial changes in DNA structure including alteration of the hydrodynamic properties of DNA [26]. It is likely such changes contribute to the enhancement of eDNA cross-linking with biopolymers and hence increases cellular aggregation.

**Figure 8:**
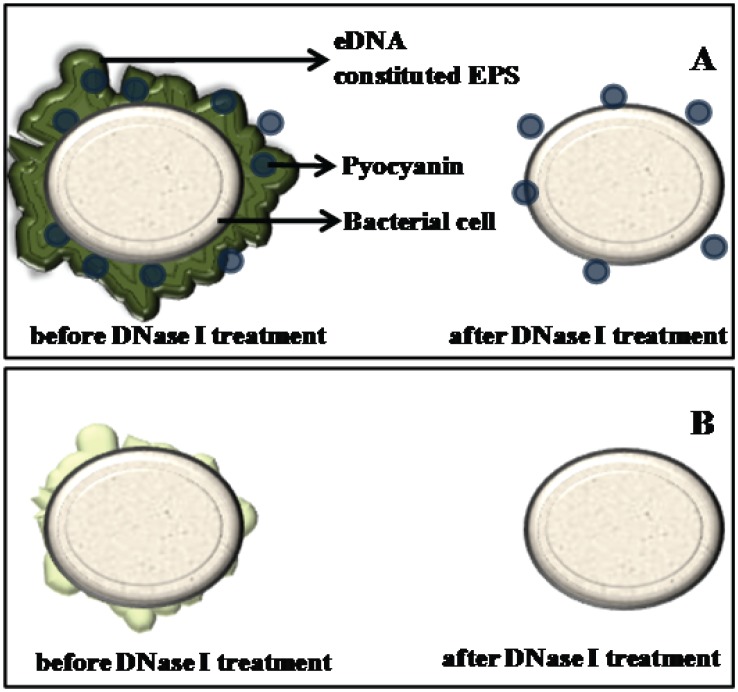
Difference in *P. aeruginosa* cell surface. (A and B) Schematic showing changes in PA14 wildtype and Δ*phzA-G* cell surface respectively before and after DNase I treatment. (A) Natural production of pyocyanin by wildtype strain facilitates EPS (mostly constitute of eDNA) binding to *P. aeruginosa* cell wall and removal of eDNA by DNase I treatment subsequently removes EPS from its cell wall. (B) The mutant Δ*phzA-G* deficient in pyocyanin production could not facilitate eDNA/EPS binding with *P. aeruginosa* cell wall, therewith no effect of DNase I treatment on its cell wall.

Supporting the above observation and argument, the hydrodynamic diameter of wildtype and Δ*phzA-G P. aeruginosa* PA14 cells showed clear variation with the size of the wildtype decreasing to the size of the pyocyanin deficient mutant after DNase I treatment (Fig. 4). This result is consistent with the suggestion that abundant biopolymers in EPS such as eDNA are anchored to the bacterial cell surface with the assistance of pyocyanin influencing surface properties such as contact angle and surface energy (Table 1). These surface properties are a reflection of the chemical composition of bacterial cell surfaces and this chemical composition drives attractive or repulsive physico-chemical interactions (Fig. 6). Without the interaction between DNA and pyocyanin the EPS layer loses stability and can be removed from bacterial cell pellets as shown in Figure 5. Figure 8 illustrates for clarity how it is envisaged that DNase I treatment affects cell surface properties of a pyocyanin producing cell in comparison to a pyocyanin deficient cell.

The competence of bacteria to bind and uptake eDNA from the extracellular environment has been well studied on various *Streptococcus* strains. In *S. intermedius* and *S. mutans* the development of competence (i.e. uptake of DNA from the environment) is mediated by a QS molecule called competence stimulating peptide (CSP) [30]. CSP triggers DNA binding and uptake, and simultaneously induces the synthesis of a DNA binding protein ComGB [31], [32] and henceforth increases bacterial aggregation and biofilm formation. Based on the data presented pyocyanin appears to play this critical role in facilitating eDNA binding to the *P. aeruginosa* cell surface and influencing its cell surface properties and aggregation.

Previous studies suggest that the production of phenazines enhances electron shuttling, antibacterial property, responsible for virulence factor in chronic lung infections, enhance eDNA production, microcolony formation and increased biomass in biofilms and eDNA is essential for biofilm stability and development of *P. aeruginosa* [6], [19], [22], [33]. We conclude that pyocyanin facilitates eDNA binding in *P. aeruginosa* and thus keeps the EPS integrated and attached to bacterial cell surfaces and consequently promotes bacterial aggregation, biofilm development and stability. Further, we propose that the thick and integrated EPS that forms due to eDNA production in association with pyocyanin is possibly shielding the bacterium in biofilms against physical and chemical challenges include shear stress, antibiotics and detergents. Production of pyocyanin and pyocyanin mediated eDNA shielding could give *P. aeruginosa* a competitive edge by protecting it from its competitor bacterial species that endure in mixed biofilms, thereby having an ecological influence in mixed biofilms. Further existence of pyocyanin along with eDNA in the sputum of cystic fibrosis (CF) patients [20], [34] could not only encourage biofilm formation but also enhance mortality in CF patients through deterioration of the host immune system. We believe this discovery may result in new strategies to combat biofilm based infections.

## References

[1] Stoodley HL, Costerton WJ, Stoodley P (2004) Bacterial biofilms: from the natural environment to infectious diseases. Nat Rev Microbiol 2: 95–108. 15040259 10.1038/nrmicro821

[2] Beech IB, Sunner J (2004) Biocorrosion: towards understanding interactions between biofilms and metals. Curr Opin Biotechnol 15: 181–186. 15193324 10.1016/j.copbio.2004.05.001

[3] Dhanasekaran D, Thajuddin N, Rashmi M, Deepika TL, Gunasekaran M (2009) Screening of biofouling activity in marine bacterial isolate from ship hull. Int J Environ Sci Tech 6: 197–202.

[4] Tsuneda S, Aikawa H, Hayashi H, Yuasa A, Hirata A (2003) Extracellular polymeric substances responsible for bacterial adhesion onto solid surface. FEMS Microbiol Lett 223: 287–292. 12829300 10.1016/S0378-1097(03)00399-9

[5] Flemming HC, Wingender J (2010) The biofilm matrix. Nat Rev Microbiol 8: 623–633. 20676145 10.1038/nrmicro2415

[6] Whitchurch CB, Tolker Nielsen T, Ragas PC, Mattick JS (2002) Extracellular DNA required for bacterial biofilm formation. Science 295: 1487. 11859186 10.1126/science.295.5559.1487

[7] Mulcahy H, Mazenod LC, Lewenza S (2008) Extracellular DNA chelates cations and induces antibiotic resistance in *Pseudomonas aeruginosa* biofilms. PLoS Pathog. 4: e1000213. 10.1371/journal.ppat.1000213 PMC2581603 19023416

[8] Das T, Sharma PK, Busscher HJ, Van der Mei HC, Krom BP (2010) Role of extracellular DNA in initial bacterial adhesion and surface aggregation. Appl Environ Microbiol 76: 3405–3408. 20363802 10.1128/AEM.03119-09 PMC2869138

[9] Das T, Krom BP, Van der Mei HC, Busscher HJ, Sharma PK (2011) DNA-mediated bacterial aggregation is dictated by acid-base interactions. Soft Matters 7: 2927–2935.

[10] Das T, Sharma PK, Krom BP, Van der Mei HC, Busscher HJ (2011) Role of eDNA on the adhesion forces between *Streptococcus mutans* and substratum surfaces: influence of ionic strength and substratum hydrophobicity. Langmuir 27: 10113–10118. 21740034 10.1021/la202013m

[11] Vilain S, Pretorius JM, Theron J, Brozel VS (2009) DNA as an adhesin: Bacillus cereus requires extracellular DNA to form biofilms. Appl Environ Microbiol 75: 2861–2868. 19251901 10.1128/AEM.01317-08 PMC2681682

[12] Huseby MJ, Kruse AC, Digre J, Kohler PL, Vocke JA, et al (2010) Beta toxin catalyzes formation of nucleoprotein matrix in staphylococcal biofilms. Proc Nat Acad Sci USA 107: 14407–14412. 20660751 10.1073/pnas.0911032107 PMC2922554

[13] Qin Z, Ou Y, Yang L, Zhu Y, TolkerNielsen T, et al (2007) Role of autolysin-mediated DNA release in biofilm formation of *Staphylococcus epidermidis* . Microbiology 153: 2083–2092. 17600053 10.1099/mic.0.2007/006031-0

[14] Thomas VC, Thurlow LR, Boyle D, Hancock LE (2008) Regulation of autolysis-dependent extracellular DNA release by *Enterococcus faecalis* extracellular proteases influences biofilm development. J Bacteriol 190: 5690–5698. 18556793 10.1128/JB.00314-08 PMC2519388

[15] Carrolo M, Frias MJ, Pinto FR, MeloCristino J, Ramirez M (2010) Prophage spontaneous activation promotes DNA release enhancing biofilm formation in *Streptococcus pneumoniae.* . PLOS ONE 5: 1–10. 10.1371/journal.pone.0015678 PMC3004956 21187931

[16] Zheng L, Chen Z, Itzek A, Ashby M, Kreth J (2011) Catabolite control protein A controls hydrogen peroxide production and cell death in *Streptococccus sanguinis* . J Bacteriol 193: 516–526. 21036992 10.1128/JB.01131-10 PMC3019840

[17] AllesenHolm M, Barken KB, Yang L, Klausen M, Webb JS, et al (2006) A characterization of DNA release in *Pseudomonas aeruginosa* cultures and biofilms. Mol Microbiol 59: 1114–1128. 16430688 10.1111/j.1365-2958.2005.05008.x

[18] Webb JS, Thompson LS, James S, Charlton T, TolkerNielsen T, et al (2003) Cell death in *Pseudomonas aeruginosa* biofilm development. J Bacteriol 185: 4585–4592. 12867469 10.1128/JB.185.15.4585-4592.2003 PMC165772

[19] Das T, Manefield M (2012) Pyocyanin promotes extracellular DNA release in *Pseudomonas aeruginosa* . PLOS ONE 7(10): e46718. 23056420 10.1371/journal.pone.0046718 PMC3466280

[20] PriceWhelan A, Dietrich LEP, Newman DK (2006) Rethinking secondary metabolism: physiological roles for phenazine antibiotics. Nat Chem Biol 2: 71–78. 16421586 10.1038/nchembio764

[21] Venkataraman A, Rosenbaum M, Arendsjan BA, Halitschke R, Angenent LT (2010) Quorum sensing regulates electric current generations of *Pseudomonas aeruginosa* PA14 in bioelectrochemical systems. Electrochem Communications 12: 459–462.

[22] Ramos I, Dietrich LEP, PriceWhelan A, Newman DK (2010) Phenazines affect biofilm formation by *Pseudomonas aeruginosa* in similar ways at various scales. Res Microbiol 161: 187–191. 20123017 10.1016/j.resmic.2010.01.003 PMC2886020

[23] Dietrich LEP, Teal TK, PriceWhelan A, Newman DK (2008) Redox-active antibiotics control gene expression and community behavior in divergent bacteria. Science 321: 1203–1206. 18755976 10.1126/science.1160619 PMC2745639

[24] Liu H, Yang Y, Shen X, Zhang Z, Shen P, et al (2008) Role of DNA in bacterial aggregation. Curr Microbiol 57: 139–144. 18491189 10.1007/s00284-008-9166-0

[25] Kholodenko AL, Douglas JF (1995) Generalised Stokes-Einstein equation for spherical particle suspensions. Phys Rev E 51: 1081–1090. 10.1103/physreve.51.1081 9962752

[26] Suh D, Chaires JB (1995) Criteria for the mode of binding of DNA binding agents. Bioorganic & Medicinal Chemistry 3: 723–728. 7582950 10.1016/0968-0896(95)00053-j

[27] van Oss CJ (1995) Hydrophobicity of biosurfaces–origin, quantitative determination and interaction energies. Colloids Surf B Biointerfaces 5: 91–110.

[28] Sharma PK, Rao KH (2003) Adhesion of *Paenibacillus polymyxa* on chal copyrite and pyrite: surface thermodynamic and extended DLVO approaches Colloids Surf B-Biointerfaces. 29: 21–38.

[29] Boks NP, Norde W, Van der Mei HC, Busscher HJ (2008) Forces involved in bacterial adhesion to hydrophilic and hydrophobic surfaces. Microbiol SGM 154: 3122–3133. 10.1099/mic.0.2008/018622-0 18832318

[30] Lappann M, Claus H, Van Alen T, Harmsen M, Ellas J, et al (2010) A dual role of extracellular DNA during biofilm formation of *Neisseria meningitides* . Mol Microbiol 75: 1355–1371. 20180907 10.1111/j.1365-2958.2010.07054.x

[31] Petersen FC, Pecharki D, Scheie AA (2004) Biofilm mode of growth of *Streptococcus intermedius* favored by a competence-stimulating signaling peptide. J Bacteriol 186: 6327–6331. 15342606 10.1128/JB.186.18.6327-6331.2004 PMC515148

[32] Petersen FC, Tao L, Scheie AA (2005) DNA binding-uptake system: a link between cell-to-cell communication and biofilm formation. J Bacteriol 187: 4392–4400. 15968048 10.1128/JB.187.13.4392-4400.2005 PMC1151753

[33] Wang Y, Wilks J, Danhorn T, Ramos I, Croal L, et al (2011) Phenazine-1-carboxylic acid promotes bacterial biofilm development via ferrous iron acquisition. J Bacteriol 193: 3606–3617. 21602354 10.1128/JB.00396-11 PMC3133341

[34] Bakker EM, Tiddens HAWM (2007) Pharmacology, clinical efficacy and safety of recombinant human DNase in cystic fibrosis. Exp Rev Respir Med 1: 317–329. 10.1586/17476348.1.3.317 20477171

